# Trends in Demographics, Hospitalization Outcomes, Comorbidities, and Mortality Risk among Systemic Sclerosis Patients

**DOI:** 10.7759/cureus.2628

**Published:** 2018-05-14

**Authors:** Oluyemisi Amoda, Virendrasinh Ravat, Sorabh Datta, Bipin Saroha, Rikinkumar S Patel

**Affiliations:** 1 College of Public Health, University of South Florida, Tampa, USA; 2 Department of Infectious Disease, Clinical Infectious Disease Specialist, Las vegas, USA; 3 Department of Internal Medicine, Providence Hospital, Washington, D.C., USA; 4 Department of Internal Medicine, University of Illinois, Chicago College of Medicine, Chicago, USA; 5 Department of Global Public Health, Arcadia University, Philadelphia, USA

**Keywords:** systemic sclerosis, comorbidity, demographics, scleroderma, mortality risk, national inpatient sample

## Abstract

Objective

This study determines trends in demographics and hospitalization outcomes among patients admitted for systemic sclerosis (SScl) and evaluates the differences between comorbidities.

Methods

The study used data from the Nationwide Inpatient Sample (NIS) for the years 2010–2014. We identified SScl as the primary diagnosis and the associated medical and psychiatric comorbidities using validated International Classification of Diseases, Ninth Revision, Clinical Modification (ICD-9­CM) codes. The differences in comorbidities and in-hospital mortality were quantified using multinomial logistic regression (odds ratio (OR)).

Results

Inpatient admissions for SScl decreased over the period 2010-2014 by 15.9% (p < 0.001). There was an increasing trend in the 61-80 years age group as they had a 29% increase in admissions and a higher risk of in-hospital mortality (OR = 2.113; p = 0.020). The differences between races showed weaker linear trends, with Caucasians (57.5%) showing an increasing trend, and African Americans (24.3%) and Hispanics (11.8%) having a decreasing trend (p < 0.001). However, Hispanics had the highest risk of mortality (OR = 1.295; p = 0.001) during hospitalization. In-hospital mortality had a linear decreasing trend, with a 10.3% decrease in deaths in 2010, and a 9.1% decrease in 2014 (p < 0.001). Hypertension (47.3%), pulmonary circulation disorders (40.1%), pulmonary fibrosis (29.7%), and congestive heart failure (24.4%) constituted the majority of comorbidities. Comorbid diabetes increased the risk of in-hospital mortality in SScl patients by four times (OR = 3.914; p = 0.003). Esophageal reflux disorder was present in only 6.7% of SScl patients, but it increased the risk of in-hospital mortality (OR = 2.643: p < 0.001). Among psychiatric comorbidities, depression (OR = 1.526; p = 0.001) and psychosis (OR = 1.743; p = 0.039) both increased the risk of in-hospital mortality.

Conclusion

We observed the various comorbidities that were associated with substantial and significant differences in the risk of in-hospital mortality. We assert that these findings indicate that comorbid conditions are influential factors that must be considered in models of health-related quality of life (HRQOL) in SScl. More attention needs to be paid to the elderly population at risk of having a higher risk of inpatient death. Further research to guide the development of clinical care models for targeting early diagnosis and treatment of comorbidities in SScl is necessary to reduce both mortality and morbidity, as well as improve the quality of care for these patients.

## Introduction

Systemic sclerosis (SScl) is a rare immune-mediated connective tissue disease characterized by vasculopathy and fibrosis of the internal organs and skin. It has a mortality greater than those of other rheumatic diseases [1]. It is associated with various comorbidities that can substantially reduce quality of life for those affected. Some of these comorbidities include thickening of the skin, ulcers of the extremities, esophageal dysmotility, pulmonary arterial hypertension, interstitial lung disease, renal crisis, and cardiac arrhythmias. It also causes functional disability, thus reducing quality of life, and has a higher negative impact on mental health relative to other medical conditions [2].

The prevalence of SScl is about 0.05%, with 85% of the cases being seen in women [3]. The disease is about five times more common in females [4] as seen in the literature and is also significantly higher among African Americans, with higher mortality rates, compared to Caucasian Americans [5]. SScl is common in the 50–58 age group [3]. Despite being a very rare disease, often described as an orphan disease, the total cost of caring for SScl per year is estimated to be USD 1.9 billion across North America. The total direct healthcare cost to an individual with SScl in the United States ranges between USD 17,000 and USD 19,000 per annum. Furthermore, the overall healthcare utilization cost increases exponentially with the involvement of the lungs [6].

There is extensive skin involvement in a majority of patients with SScl, and this forms the basis of the classification of the disease. Those with proximal skin involvement are said to have diffuse sclerosis, while those with skin involvement of the distal to the elbows and the knees with/without face and neck involvement are said to have limited disease [1, 7]. The two classes/subsets of the disease have a distinct pattern of organ involvement and specific serological markers. There is also a small group of people suffering from the disease without skin manifestations, called visceral scleroderma or systemic sclerosis sine scleroderma [1, 8].

The effect of SScl on the lungs can either manifest as parenchymal causing interstitial lung disease or as vascular causing pulmonary hypertension. A small set of people with SScl may also be affected by secondary pulmonary disease resulting from gastroesophageal reflux disease (GERD), toxicity, infections, and malignancy. Pulmonary complications caused by SScl are now the main cause of death resulting from the disease [9-10]. Earlier, renal crisis used to be the highest cause of mortality resulting from the disease, mainly affecting people with diffuse disease.

To the best of our knowledge, this is the first study to evaluate the inpatient prevalence of SScl and trends in demographics and hospitalization outcomes with a comparison of the risk of mortality associated with various comorbidities.

## Materials and methods

Data source

We used Nationwide Inpatient Sample (NIS) data from the Healthcare Cost and Utilization Project (HCUP) sponsored by the Agency for Healthcare Research and Quality (AHRQ) [11]. HCUP combines data from state organizations, hospital associations, private data organizations, and the federal government to create a national information resource consisting of patient-level healthcare data. The NIS database has inpatient data from over 1,000 hospitals across 44 states in the United States.

Sample population

We identified all discharges for which an International Classification of Diseases, Clinical Modification, Ninth Revision (ICD-9-CM) code of 710.1 was included in the discharge diagnoses, thereby identifying the patients with diffuse systemic sclerosis, but excluding those with localized scleroderma. We also excluded all patients with ICD-9-CM codes for systemic lupus erythematosus (SLE; 710.0) or rheumatoid arthritis (RA; 714.0) in order to improve the reliability for a diagnosis of SScl. Population hospitalization rates for SScl patients were determined for the years 2010-2014 and organized by age group, gender, and race. Ages were divided into five groups: 1–20, 21–40, 41–60, 61–80, and >80 years; races were classified as Caucasian, African American, Hispanic, Asian, Native American, and others.

Assessment of inpatient outcomes

To measure the differences in hospitalization outcomes in SScl patients over the period 2010-2014, the outcome variables of interest included severity of illness, which measures the loss of body functions, in-hospital mortality, which measures the number of inpatient deaths, and disposition of the patient [11]. We calculated the length of inpatient stay as the number of nights the patient remained in the hospital for SScl. Total charges of hospitalization do not include professional fees and non-covered charges. If the source provided total charges with professional fees, then the professional fees were removed from the charge during NIS data processing.

Assessment of comorbidities

Comorbidities are considered as coexisting conditions to SScl and are the disorders under study. AHRQ comorbidity software was utilized to generate binary variables [12]. Using ICD-9-CM diagnosis codes, this variable identified 14 comorbidities in the discharge records. As shown in Table 1, ICD-9-CM codes were used to tag comorbidities.

**Table 1:** ICD-9-CM diagnosis codes for comorbidities associated with systemic sclerosis The variables are Agency for Healthcare Research and Quality (AHRQ) comorbidity measures.

Comorbidity	ICD-9-CM Diagnosis Codes
Pulmonary Circulation Disorder	415.11-415.19, 416.0-416.9, 417.9
Pulmonary Hypertension	416.0
Pulmonary Fibrosis	516.31
Cardiac Arrhythmia	427.0, 427.1, 427.2, 427.31, 427.32, 427.60, 427.61, 427.69, 427.81, 427.89, 427.9, 785.0, 785.1
Congestive Heart Failure	398.91, 402.01, 402.11, 402.91, 404.01, 404.03, 404.11, 404.13, 404.91, 404.93, 428.0-428.9
Diabetes without Complication	249.00-249.31, 250.00-250.33, 648.00-648.04
Diabetes with Complication	249.40-249.91, 250.40-250.93, 775.1
Renal Failure	403.01, 403.11, 403.91, 404.02, 404.03, 404.12, 404.13, 404.92, 404.93, 585.3, 585.4, 585.5, 585.6, 585.9, 586, V42.0, V45.1, V45.11, V45.12, V56.0-V56.32, V56.8
Hypertension	401.1, 401.9, 642.00-642.04, 401.0, 402.00-405.99, 437.2, 642.10-642.24, 642.70-642.94
Liver Diseases	070.22, 070.23, 070.32, 070.33 , 070.44, 070.54, 456.0, 456.1, 456.20, 456.21, 571.0, 571.2, 571.3, 571.40- 571.49, 571.5, 571.6, 571.8, 571.9, 572.3, 572.8, 573.5, V42.7
Gastroesophageal Reflux Disease	530.81
Depression	300.4, 301.12, 309.0, 309.1, 311
Psychosis	295.00-298.9, 299.10, 299.11
Neurological Disorder	330.1-331.9, 332.0, 333.4, 333.5, 333.71, 333.72, 333.79, 333.85, 333.94, 334.0-335.9, 338.0, 340, 341.1-341.9, 345.00-345.11, 345.2-345.3, 345.40-345.91, 347.00-347.01, 347.10-347.11, 649.40-649.44, 768.7, 768.70, 768.71, 768.72, 780.3, 780.31, 780.32, 780.33, 780.39, 780.97, 784.3

Statistical analysis

The population estimated in this study is derived from the NIS database. We have adjusted the means, proportions, and standard errors of the discharge weights. We also used Pearson’s chi-square (χ2) test and the independent sample T-test for verifying and validating the continuous data, respectively. Furthermore, the categorical variables are in percentages. We also used discharge weight, which is given in the NIS database, to obtain national inpatient data. A p-value lesser than 0.05 was used to determine the statistical significance of the test result. Differences in comorbidities were quantified using chi-square tests. We used a multinomial logistic regression model to quantify associations between the comorbidities and in-hospital mortality (odds ratio (OR) and 95% confidence interval (CI)). Statistical analysis was also done using Statistical Package for the Social Sciences (SPSS) version 23 (IBM Corporation, New York) [13]. Our database did not contain any patients' personally identifiable information. Therefore, institutional review board (IRB) approval was not required for this study.

## Results

Trends in demographic characteristics

The total number of admissions with primary diagnosis of SScl over the period 2010 to 2014 was 8,851. The proportion of admissions involving SScl saw a decreasing trend (p < 0.001) from 1825 (in 2010) to 1535 (in 2014), representing a 15.9% decrease over five years.

The overall mean age of SScl patients (55.76 ± 15.718 years) increased by 1.95 years from 2010-2014 (p < 0.001). This is due to decreasing trends in younger age groups (ages < 60 years) and increasing trends in the older age group of 61-80 years, which resulted in a 29% increase over the five years. SScl was predominant in females (80.5%) with an increasing trend, but the result was not statistically significant (p = 0.103). Differences between races showed weaker linear trends, with Caucasians (57.5%) and Asians/Pacific Islanders (2.4%) having an increasing trend, and African Americans (24.3%) and Hispanics (11.8%) having a decreasing trend (p < 0.001).

Close to half of all the patients with SScl had Medicare as the primary source of health insurance coverage, which is in keeping with the norm in that age group, with a range between 42.3% and 46.9%. Private insurance was the second major source of payment accounting for 29.4% to 33.2% of payments over a period of five years. In terms of median household income levels, the highest quartiles, i.e., patients with 75th-100th percentile median household income, showed a weak linear increasing trend, and the number of patients from the 26th-50th percentile income group increased by 14.5% (p < 0.001). The demographic trends in inpatient SScl admissions is mentioned in Table 2.

**Table 2:** Demographic trends in systemic sclerosis (SScl) in the inpatient population Significant p-values ≤ 0.05 at 95% confidence interval.

Variable	2010	2011	2012	2013	2014	Total	p-value	Trend Direction
Number of admissions	1825	2026	1800	1665	1535	8851	< 0.001	Decreasing
Age								
Mean age	54.01	55.96	56.24	56.73	55.96	55.76	< 0.001	Increasing
1-20 years	2.6 %	1.2 %	4.4 %	2.4 %	2.6 %	2.6 %	< 0.001	Stable
21-40 years	15.7 %	14.7 %	8.9 %	11.4 %	14.3 %	13.0 %		Decreasing
41-60 years	46.7 %	43.6 %	45.0 %	44.4 %	39.7 %	44.0 %		Decreasing
61-80 years	30.3 %	36.5 %	37.5 %	39.3 %	39.1 %	36.4 %		Increasing
> 80 years	4.7 %	4.0 %	4.2 %	2.4 %	4.2 %	3.9 %		Decreasing
Gender								
Male	20.3 %	20.7 %	19.2 %	19.5 %	17.3 %	19.5 %	0.103	Decreasing
Female	79.7 %	79.3 %	80.8 %	80.5 %	82.7 %	80.5 %		Increasing
Race								
Caucasian	51.9 %	62.0 %	56.9 %	60.7 %	55.6 %	57.5 %		Increasing
African American	27.7 %	24.5 %	25.8 %	23.0 %	19.8 %	24.3 %		Decreasing
Hispanic	14.9 %	7.4 %	10.0 %	12.8 %	14.9 %	11.8 %		Decreasing
Asian/Pacific Islander	2.7 %	1.9 %	2.9 %	0.6 %	3.8 %	2.4 %		Increasing
Native American	1.2 %	0.6 %	1.2 %	0.6 %	0.7 %	0.9 %		Decreasing
Other	1.5 %	3.6 %	3.2 %	2.2 %	5.2 %	3.1 %		Increasing
Insurance								
Medicare	42.3 %	49.4 %	46.4 %	49.2 %	46.9 %	46.9 %	< 0.001	Increasing
Medicaid	18.3 %	9.8 %	13.9 %	12.9 %	13.0 %	13.5 %		Decreasing
Private insurance	29.4 %	33.1 %	31.7 %	31.5 %	33.2 %	31.8 %		Variable
Self-pay	5.7 %	5.3 %	4.4 %	2.4 %	3.3 %	4.3 %		Decreasing
No charge	0.7 %	0.5 %	0.0 %	0.3 %	0.3 %	0.4 %		Decreasing
Other	3.7 %	1.9 %	3.6 %	3.6 %	3.3 %	3.2 %		Decreasing
Median Household Income								
0-25th percentile	32.2 %	25.1 %	30.4 %	29.5 %	30.2 %	29.3 %	< 0.001	Decreasing
26th-50th percentile	25.5 %	23.3 %	22.2 %	24.9 %	29.2 %	24.9 %		Increasing
51st-75th percentile	21.2 %	25.5 %	19.9 %	25.2 %	19.9 %	22.5 %		Decreasing
76th-100th percentile	21.1 %	26.2 %	27.6 %	20.3 %	20.6 %	23.4 %		Increasing

Elderly SScl patients had a higher risk of in-hospital mortality as seen in Table 3. Females did not show a higher risk of mortality when compared to males. Furthermore, among all races, Hispanics had the highest risk of mortality during hospitalization for SScl.

**Table 3:** Demographic predictors of in-hospital mortality in systemic sclerosis patients Significant p-values ≤ 0.05 at 95% confidence Interval.

Demographic Predictors	Odds Ratio	95 % Confidence Interval	p-value
Age			
1-20 years	Referent		
21-40 years	1.017	0.517 – 1.997	0.962
41-60 years	1.524	0.811 – 2.864	0.191
61-80 years	2.113	1.124 – 3.971	0.020
> 80 years	2.745	1.358 – 5.550	0.005
Gender			
Male	Referent		
Female	0.893	0.734 – 1.087	0.260
Race			
Caucasian	Referent		
African American	0.775	0.624 – 0.963	0.021
Hispanic	1.295	1.012 – 1.656	0.040
Asian/Pacific Islander	0.916	0.529 – 1.587	0.755
Native American	< 0.001		0.997
Other	1.876	1.294 – 2.719	0.001

Trends in hospitalization outcomes

About 86.2% SScl patients were admitted based on an emergency condition, while 13.8% were admitted on an elective basis (p < 0.001). SScl patients with severe morbidity made up 71.4% of the total admissions over the five-year period and showed a linear decreasing trend from 76% (in 2010) to 70.4% (in 2014) (p < 0.001). The mean length of inpatient stay was 8.6 days and had a weak linear increasing trend from 9.1 days in 2010 to 9.4 days in 2014. However, total inpatient charges had a strong linear increasing trend (p < 0.001), with the overall mean inpatient charge adding up to USD 82,520. In-hospital mortality also had a linear decreasing trend with 10.3% deaths in 2010 and 9.1% in 2014 (p < 0.001). Further hospitalization trends in SScl inpatient admissions are described in Table 4.

**Table 4:** Hospitalization trends in systemic sclerosis in the inpatient population Significant p-values ≤ 0.05 at 95% confidence interval. USD: United States Dollars; SNF: Skilled nursing facility; INF: Intermediate nursing facility; HHC: Home Health Care; AMA: against medical advice

Variable	2010	2011	2012	2013	2014	Total	p-value	Trend
Admission Type								
Non-elective	86.3 %	86.1 %	83.0 %	87.3 %	88.6 %	86.2 %	< 0.001	Increasing
Elective	13.7 %	13.9 %	17.0 %	12.7 %	11.4 %	13.8 %		Decreasing
Severity of Illness / Morbidity								
Minor	1.9 %	3.6 %	3.6 %	6.0 %	3.6 %	3.7 %	< 0.001	Increasing
Moderate	21.7 %	21.1 %	30.3 %	25.8 %	26.1 %	24.9 %		Variable
Major	76.0 %	75.3 %	66.1 %	68.2 %	70.4 %	71.4 %		Decreasing
Inpatient Stay (in days)								
Mean stay	9.1	7.8	8.5	8.5	9.4	8.6	< 0.001	Variable
Inpatient Total Charge (in USD)								
Mean charge	69713	77131	80095	86080	103884	82520	< 0.001	Increasing
In-hospital Mortality								
Did not die	89.7 %	94.3 %	92.8 %	91.6 %	90.9 %	91.9 %	< 0.001	Increasing
Died	10.3 %	5.7 %	7.2 %	8.4 %	9.1 %	8.1 %		Decreasing
Disposition / discharge								
Routine	57.4 %	60.1 %	58.9 %	53.5 %	55.0 %	57.2 %	< 0.001	Decreasing
Short-term hospital	4.9 %	2.1 %	3.1 %	3.0 %	3.3 %	3.3 %		Decreasing
SNF/INF	9.8 %	14.1 %	10.8 %	13.2 %	12. 1%	12.0 %		Increasing
HHC	17.0 %	17.0 %	19.4 %	21.3 %	19.9 %	18.8 %		Increasing
AMA	0.6 %	1.0 %	0.6 %	0.3 %	0.7 %	0.6 %		Variable

Trends in comorbidities

Hypertension (47.3%), pulmonary circulation disorders (40.1%), pulmonary fibrosis (29.7%), and congestive heart failure (24.4%) constituted the majority of these comorbidities. Among these comorbidities, heart failure, hypertension, and pulmonary circulation disorders showed an increasing linear trend (p < 0.001, < 0.001 and p = 0.026; respectively). On the contrary, pulmonary fibrosis had a decreasing trend (decreased by 21%, p < 0.001) over five years. Depression increased from 13.1% to 15.6% from 2010-2014 (p < 0.001), with an overall increase of 19%. Of note, diabetes without chronic complications had an 18.75% increase in the five-year period, with a strong increasing trend (p < 0.001). Other chronic conditions such as cardiac arrhythmia, renal failure, gastroesophageal reflux disease (GERD), and liver disease also showed weak linear increasing trends as mentioned in Table 5.

**Table 5:** Comorbidities trends in systemic sclerosis in the inpatient population Significant p-values ≤ 0.05 at 95% confidence interval. Variables are Agency for Healthcare Research and Quality (AHRQ) co-morbidity measures.

Comorbidity	2010	2011	2012	2013	2014	Total	p-value	Trend
Pulmonary Circulation Disorder	39.8 %	38.3 %	43.3 %	39.6 %	39.4 %	40.1 %	0.026	Increasing
Pulmonary Hypertension	0.6 %	1.2 %	0.8 %	0.9 %	1.3 %	1.0 %	0.212	Increasing
Pulmonary Fibrosis	34.7 %	27.4 %	32.8 %	25.8 %	27.4 %	29.7 %	< 0.001	Decreasing
Cardiac Arrhythmia	6.1 %	6.1 %	5.3 %	5.1 %	7.5 %	6.0 %	0.038	Increasing
Cardiac Heart Failure	23.7 %	23.3 %	27.8 %	23.4 %	23.8 %	24.4 %	0.007	Increasing
Diabetes without Complication	9.6 %	9.6 %	7.8 %	10.8 %	11.4 %	9.8 %	0.005	Increasing
Diabetes with Complication	2.0 %	1.2 %	1.4 %	3.3 %	0.3 %	1.6 %	< 0.001	Decreasing
Renal Failure	15.5 %	15.3 %	14.4 %	17.7 %	16.3 %	15.8 %	0.097	Increasing
Hypertension	43.0 %	47.7 %	50.6 %	54.1 %	41.0 %	47.3 %	< 0.001	Increasing
Liver Diseases	3.7 %	4.2 %	3.6 %	3.9 %	4.2 %	3.9 %	0.808	Increasing
Gastroesophageal Reflux Disease	4.9 %	6.3 %	6.7 %	9.3 %	6.5 %	6.7 %	< 0.001	Increasing
Depression	13.1 %	18.1 %	13.9 %	16.2 %	15.6 %	15.4 %	< 0.001	Increasing
Psychosis	2.2 %	4.4 %	1.4 %	4.2 %	4.2 %	3.3 %	< 0.001	Increasing
Neurological Disorder	6.3 %	6.5 %	6.9 %	6.9 %	5.5 %	6.5 %	0.474	Decreasing

Comorbid diabetes with chronic complications increased the risk of in-hospital mortality in SScl patients by four times (OR = 3.914; 95% CI 1.583 – 9.677; p = 0.003). GERD was present in 6.7% of SScl patients, but it increased the risk of in-hospital mortality by 2.6 times (OR = 2.643; 95% CI 1.566 – 4.460; p < 0.001). Among psychiatric comorbidities, depression (OR = 1.526; 95% CI 1.192 – 1.954; p = 0.001) and psychosis (OR = 1.743; 95% CI 1.030 – 2.951; p = 0.039) increased the risk of in-hospital mortality. In-hospital mortality risk in SScl patients due to medical and psychiatric comorbidities is shown in Table 6.

**Table 6:** In-hospital mortality risk in systemic sclerosis patients by comorbidities Significant p-values ≤ 0.05 at 95% confidence interval. Variables are Agency for Healthcare Research and Quality (AHRQ) co-morbidity measures.

Comorbidity	Odds Ratio	95 % Confidence Interval	p-value
Pulmonary Circulation Disorder	0.288	0.254 – 0.327	< 0.001
Pulmonary Hypertension	1.862	0.742 – 4.675	0.186
Pulmonary Fibrosis	1.166	0.968 – 1.405	0.106
Cardiac Arrhythmia	1.094	0.780 – 1.536	0.602
Cardiac Heart Failure	0.561	0.472 – 0.665	< 0.001
Diabetes without Complication	1.230	0.933 – 1.621	0.142
Diabetes with Complication	3.914	1.583 – 9.677	0.003
Renal Failure	0.510	0.419 – 0.621	< 0.001
Hypertension	1.331	1.123 – 1.577	0.001
Liver Diseases	0.626	0.453 – 0.865	0.005
Gastroesophageal Reflux Disease	2.643	1.566 – 4.460	< 0.001
Depression	1.526	1.192 – 1.954	0.001
Psychosis	1.743	1.030 – 2.951	0.039
Neurological Disorder	0.919	0.676 – 1.250	0.591

## Discussion

There was a decreasing trend in the number of admissions for SScl from 2010 to 2014, which may be due to advances in the treatment of the disease with the use of immunosuppressive agents and anti-fibrotic tyrosine kinase inhibitors to help control the pulmonary complications of the disease [14]. This may also be due to greater recognition of the disease by primary care physicians and quicker referral of the patients to rheumatologists who are then able to make the correct diagnosis and institute better management and follow up on an outpatient basis, thereby preventing unnecessary complications requiring admission [15].

The demographics share a similar trend to those from previous studies, especially the data on age and gender. Contrary to other studies which showed a higher prevalence of the disease in those identifying as Black [3], our study shows a higher prevalence among Caucasians (52.7%) admitted to hospitals compared to African Americans (24%) over the five-year period. However, Hispanics had a higher risk of mortality during hospitalization, compared to the other races. This may be due to the socioeconomic and health insurance status of the patients. The decreasing trend seen in patients less than 60 years of age may be because of a greater awareness in people of that age group, or a high index of suspicion amongst primary care physicians who are quickly able to recognize the symptoms [15]. The increasing trend in admissions of patients between ages 61 and 80 can be credited to a lower index of suspicion in these patients and the presence of other comorbidities that may mask the symptoms or to which the symptoms may be attributed. However, a study showed that SScl affects age groups differently depending on race, with African Americans affected at an earlier age compared to Caucasians [16]. As mentioned in our results, Medicare was the most common type of insurance for patients with SScl that were admitted, in line with the norm in the age group that is mostly affected. This is followed by people with private insurance, correlating with the working age group, who have employer-sponsored insurance.

As with most chronic medical conditions, 85% of SScl patients who were admitted to the hospital over the five-year study period were admitted through the emergency department due to either complications or the worsening of their condition, requiring emergent care. In keeping with the debilitation caused by the disease [2], over 70% had severe disease that limited body functions. There was a variable trend in mean inpatient stay of 9.1 days in 2010 and 9.4 days in 2014, and the mean inpatient charges increased by 18% over these five years. With greater innovations in treatment, more SScl patients were discharged home following an admission compared to those discharged to a short-term hospital or nursing facility. This indicates that they do not need skilled or immediate nursing care following an admission, suggesting early recognition of complications and better collaboration between primary care physicians and other specialists in the coordination of care [15].

Consistent with the age of most SScl patients in this cohort, there were some chronic medical conditions that were associated with the disease. These conditions, including hypertension, pulmonary circulation disorders, diabetes, and pulmonary fibrosis are already more common in these age groups. A limitation of the study is the inability to determine the sequence of SScl and the comorbidities. It is unknown if the disease is predisposed to these conditions or if it made the conditions worse. There was a decreasing trend over the years for pulmonary fibrosis, which shows that there has been more effective treatment [14]. Diabetes rates also increased over the five-year period, which may be a side effect of steroids used in the management of the disease. The rate of depression in SScl patients increased by 19% between 2010 and 2014, although the overall rates over five years were considerably lower (13%-15%) than those seen in a study about depression in SScl, which took place in Italy (65%) [17]. A study done in the United States about the mental health of SScl patients showed that 6% of the patients studied had depression, while 25% were diagnosed with generalized anxiety disorder [18]. This trend seems to reveal the fact that the mental health of the patients affected has still not been addressed appropriately. The enormity of the disease can have serious psychological effects on patients, which need to be dealt with early in the disease through counselling, although most patients develop coping and defense mechanisms to deal with the emotional stress that accompanies the disease [18].

A study done to evaluate mortality resulting from SScl between 1999 and 2001 showed a crude mortality rate of 4.7, with women 3.2 times more likely to die from the disease, and higher death rates seen amongst African American patients [5]. Previous studies also showed that patients with SScl are more likely to die from complications such as pulmonary hypertension and renal crisis. In this study, we found that the overall mortality in admitted SScl patients was 8.75% and that there was a 21% decline in in-hospital mortality from 2010 to 2014. Mortality was five times higher in patients with comorbid complicated diabetes although we were unable to deduce from the data the primary causes of these deaths. Our study did not appreciate any differences in the association with in-hospital mortality based on gender.  

NIS is an administrative database and lacks the patient-level data needed to make accurate clinical associations. Furthermore, these types of retrospective studies are always subject to selection bias, which might be accentuated by the moderate sensitivity of diagnostic codes for SScl and associated medical and psychiatric comorbidities. We also could not account for re-hospitalizations, given the nature of the database, although they add to the total inpatient burden. Despite these limitations, the NIS database provides an unparalleled population-based perspective on disease associations with systematic and temporal factors and provides a rationale for further in-depth studies. This dataset is subject to minimal reporting bias, and all information is coded independently of the individual practitioner, making it a potentially more reliable source.

## Conclusions

Among an inpatient sample of SScl patients, we observed various medical and psychiatric comorbidities that were associated with substantial and significant differences in the risk of in-hospital mortality. We assert that these findings indicate that comorbid conditions are influential factors that must be considered in models of health-related quality of life (HRQOL) in SScl. More attention needs to be paid to the elderly population that is at a higher risk of inpatient death. There is also a rising trend in many comorbid conditions over the period of 2010-2014 that are associated with a marked increase in total inpatient charges, which needs to be addressed. Further research to guide the development of clinical care models for targeting early diagnosis and treatment of comorbidities in SScl is necessary to both reduce mortality and morbidity and improve quality of care in these patients.
